# Editorial Note: MiR172-*APETALA2-like* genes integrate vernalization and plant age to control flowering time in wheat

**DOI:** 10.1371/journal.pgen.1011544

**Published:** 2025-01-06

**Authors:** The PLOS Genetics Editors

This Editorial Note is issued to provide an update to the Expression of Concern notice previously issued on this article [1, 2].

At the time the interim Expression of Concern notice was issued [2], the authors were in the process of repeating the experiments underlying the results presented in Figs 6 and 7. Due to the time needed to conduct and review these experiments, the replication data were not included with the Expression of Concern notice [2] and are being provided in this follow-up notice. Information on the repeat experiments and how these data affect the published results is available with this notice. The results of repeat experiments were assessed by a reviewer of the original article, who concluded that the repeat experiment data are consistent with the original study and that the overall conclusions stand.

Specifically, the authors repeated the qRT-PCR experiments presented in Figs 6 and 7 and re-evaluated heading time and leaf number using samples from new plant material. The supporting data for the modified Figs 6 and 7 are provided in the S1 File available with this notice.

The authors state that the major conclusions reported in the original study remain unaffected, but that the replicate experiments highlighted some differences in the expression results reported in the originally published Figs 6 and 7 (see S2 File) and corresponding Results and Discussion sections. In addition, the authors discovered that they erroneously calculated days to heading data for the non-vernalized plants (originally published Figs 6A and 7A, panel NV), using the germination day instead of the day when the NV plants were at the same developmental stage as the other plants at the end of the vernalization treatments. This resulted in the incorrect addition of 18 d to the heading time of the NV plants). In the updated data set provided in the S1 File and in Figs 6A and 7A, the 18 d were subtracted for all genotypes in the NV panels. This change does not affect the statistical comparisons among genotypes within the NV treatment.

The specific changes to the article’s results are described in Table 1 below.

**Table 1:** Summary of changes to the results.

**Fig**	**Previously published data (error)**	**Revised heading time (based on corrected error)**
6A & 7A	Heading times of the non-vernalized plants (NV) were recorded from germination to heading, whereas heading times of the vernalized plants were recorded from the time they were removed from vernalization.	To correct this inconsistency, the 18 d (from germination to the time when the other vernalized plants were removed from vernalization) were subtracted from the heading times of all four genotypes in the NV plants.
**Fig**	**Previously published data (manipulated)**	**Revised data (based on repeated experiments)**
6C	Comparisons of leaf miR172 levels between winter Kronos and *vrn1-null* plants vernalized for 6 weeks (w) showed significantly higher expression levels of miR172 in the plants with the functional *VRN1* allele.	Comparisons of leaf miR172 levels between winter Kronos and *vrn1-null* plants vernalized for 6 w showed a slight, but not significant, increase in miR172 expression in plants with the functional *VRN-B1* winter allele. Other comparisons were consistent between both experiments.
6D & 6E	Reduced miR172 levels in MIM172 plants resulted in significant increases in the transcript levels of *AP2L5* relative to other genotypes in all treatments and of *AP2L1* in the NV and 3w vernalization treatments only.	Reduced miR172 levels in MIM172 plants resulted in significant increases in the transcript levels of *AP2L5* relative to other genotypes in all treatments, while *AP2L1* levels showed non-significant differences.
6F, 6G & 6H	After 3 w vernalization, winter *UBI*_*pro*_:*miR172* plants expressed significantly higher levels of *FT1* and *VRN1* and lower levels of *VRN2* than the other genotypes. After 6 w vernalization, *VRN1* and *FT1* were significantly upregulated and *VRN2* significantly downregulated in, winter Kronos, MIM172 and *UBI*_*pro*_:*miR172* relative to *vrn1-null*. For *FT1*, *UBI*_*pro*_:*miR172* was significantly higher than winter and MIM172.	After 3 w vernalization, winter *UBI*_*pro*_:*miR172* plants expressed significantly higher levels of *FT1* than the other genotypes, except winter Kronos. *VRN1* and *VRN2* showed no significant differences among genotypes. After 6 w vernalization, *VRN1* and *FT1* were significantly upregulated and *VRN2* significantly downregulated in winter Kronos, MIM172 and *UBI*_*pro*_:*miR172* relative to *vrn1-null*. For *FT1*, *UBI*_*pro*_:*miR172* was not significantly different from winter and MIM172.
7C-H	The non-vernalized winter *ap2l1*, and *ap2l1 ap2l5* mutant plants showed significantly higher levels of *VRN1* and *FT1* than the winter control. These differences were maintained after 2 w vernalization but were significant only for *FT1*. *VRN2* expression was high in both treatments and differences among genotypes were either not significant (in NV) or significant only between *UBI*_*pro*_:*miR172* and *ap2l5*.	In plants vernalized for 2 w or not vernalized, *VRN*-*B1* and *FT1* transcript levels in the 9^th^ leaf were very low and not significantly different among genotypes. *VRN2* expression was high in both treatments and differences among genotypes were either not significant (2 w vernalization) or significant only between *ap2l1* and both winter and *ap2l5* (NV). An additional 2 w vernalization experiment was added to Fig 7 (panels F-H). Consistent with their earlier heading time, the *ap2l1 ap2l5* combined mutant and the *UBI*_*pro*_:*miR172* transgenic plants showed significantly higher levels of *VRN-B1* and *FT1* and lower levels of *VRN2* than the winter control plants in the 15^th^ leaf. No significant differences among genotypes were detected in the 12^th^ leaf.

The small differences in expression described above do not affect any of the critical differences among genotypes or any of the conclusions reported in this manuscript.

In light of these differences this article [1] was republished on December 13, 2024, to update Figs 6 and 7, and multiple paragraphs of the respective Results and Discussion sections discussing the Fig 6 and Fig 7 results. Please download this article again to view the correct version. The original version of the published article remains available in the S2 File provided with this notice. The S3 File highlights the changes made to the article’s text at the time of republication.

The PLOS Publication Ethics team reviewed this case in collaboration with the *PLOS Genetics* Editors, and carefully considered case details including the confirmed data manipulation, the observation that the data in question comprise a relatively minor portion of the results, and the reliability of the article’s main findings as demonstrated by the repeat data and supported by an Editorial Board member. PLOS issues this Editorial Note to inform readers that the Expression of Concern [2], issued previously to inform readers of the data manipulation concerns and that findings in the originally published Figs 6 and 7 (see S2 File) are unreliable, is final. However, the journal stands by the article once amended with this notice to include these alerts and the data obtained in repeat experiments.

## Supporting information

S1 File Underlying data for the replicate experiments presented in the updated Figs 6 and 7. (ZIP)

S2 File Originally published PDF of this article [1]. (PDF)

S3 File Manuscript with tracked changes to highlight updates to results and discussion section following repeat experiments. (DOCX)
