# Supplementary figures and images for: Editorial Note: MiR172-APETALA2-like genes integrate vernalization and plant age to control flowering time in wheat
Source: PLoS Genet. 2025 Jan 6;21(1):e1011544. doi: 10.1371/journal.pgen.1011544 (PMC11703051; doi:10.1371/journal.pgen.1011544)

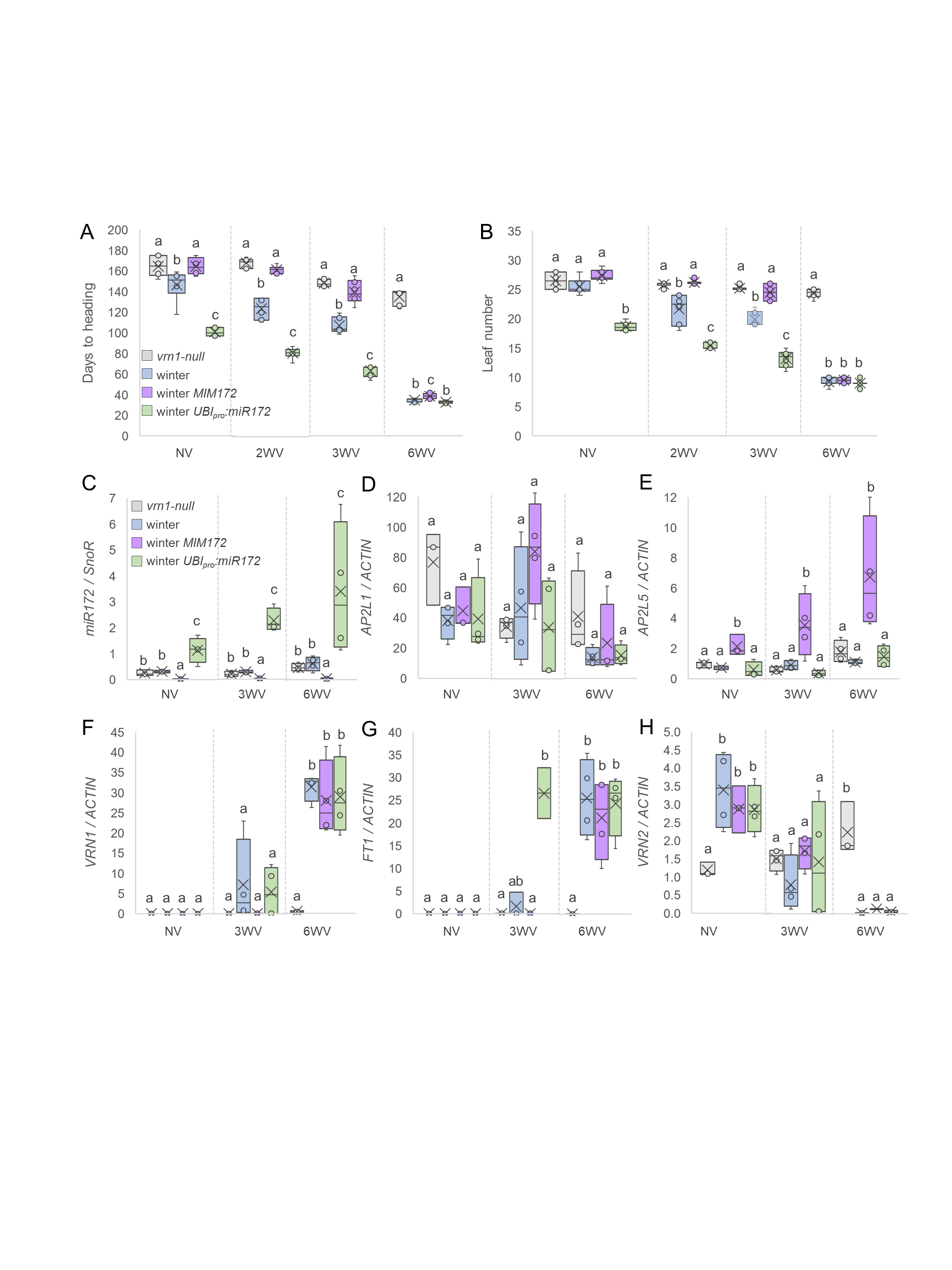

Supplement: S1 File — (ZIP) [file pgen.1011544.s001.zip › S1 File pgen.1010157 - repeat experiment data/Fig 6 R2.tif]

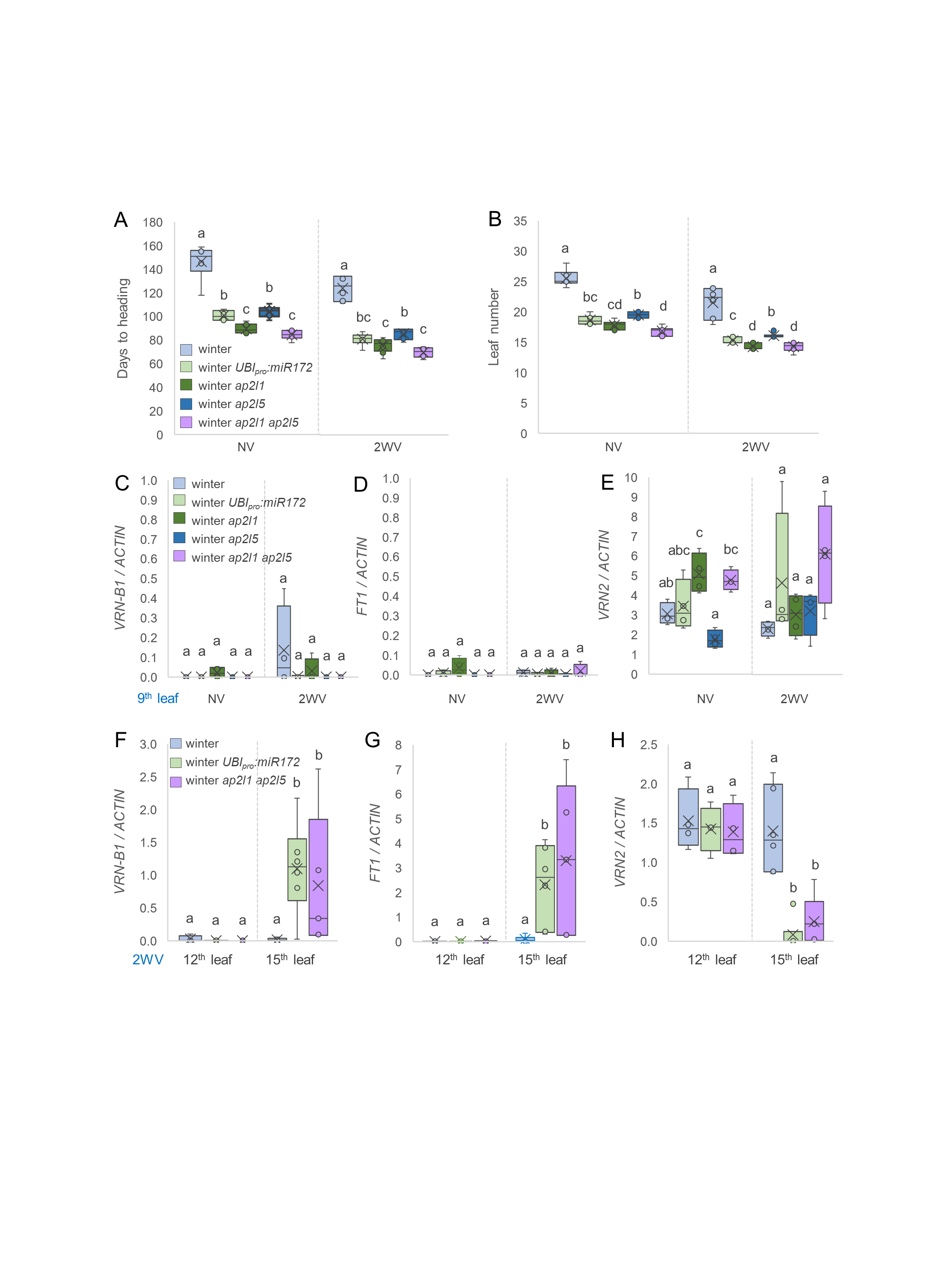

Supplement: S1 File — (ZIP) [file pgen.1011544.s001.zip › S1 File pgen.1010157 - repeat experiment data/Fig 7 R2.tif]
